# Supplementary material for: Ageing in place or stuck in place: Preferred care setting for community-dwelling older persons in a low-resource country in Sub Saharan Africa
Source: PLoS One. 2023 Oct 16;18(10):e0292939. doi: 10.1371/journal.pone.0292939 (PMC10578603; doi:10.1371/journal.pone.0292939)
Supplement: Attachment — Submitted filename: Response to Reviewers Comments_20230312.docx [file pone.0292939.s001.docx]

Dear Sir,

Thank you for the detailed review of my manuscript. Please find below the response to the comments made.

Thank you.

E. Cadmus

| **Serial Number** | **Comment** | **Response** |
| --- | --- | --- |
|  | **Reviewer 1** |  |
| 1. | **Abstract**  - The objective presented refers to the qualitative study and the method only refers to what was done at a qualitative level. Here it would be important to give a broader view of the study, including the quantitative study, because in this way the idea of the mixed study is lost. | ‘This study determined factors associated with the preferred care setting among community-dwelling older persons (quantitative objective) and explored their views about their choices (qualitative objective)  Line 18-19. |
|  | - As for the results, I suggest that you present the categories and sub-categories within each theme and write one or two sentences that give an overview of the results. | Done (Table 4) |
|  | **Introduction** |  |
|  | The expression “older populace”, which appears for the first time in line 62 and then is repeated throughout the article, should be replaced by the expression older/elderly population | Revised (Line 58) |
|  | The abbreviation QoL appears for the first time on line 80, so it must be spelled out in full. | Revised (Line 82) |
|  | In lines 87 and 88, the references are written in full instead of being numbered. The referencing must be consistent throughout | Revised (Line 89 and 91) |
|  | **Introduction** |  |
|  | - The Introduction ends with the objective of the study, but it could be clearer. I suggest that objectives be formulated for qualitative and quantitative studies and that the articulation between the two be clarified. | This study compared using quantitative measures the preferred care setting for ageing and factors associated with the decision among rural and urban community-dwelling older persons aged 60 years and above in Oyo State, Nigeria. Also, the study explored using qualitative measures the views about their choices  Revised ( Line140-143) |
|  | **Materials and methods** |  |
|  | It would be important to start this section with the justification for choosing the mixed methodology. I suggest that the articulation between the data from the quantitative and qualitative studies be clearly explained and also clearly explained what the second study (which it was also necessary to say) added to the first. | Revised ( Line 146-157) |
|  | This section mentions that the methodology has already been described previously in other studies that are referenced, but I suggest that the methodology is also presented here, albeit briefly. This so that the reader does not have to resort to other articles to understand what was done in this one. | Revised ( Line 158) |
|  | Methodological procedures should be clearer and more complete, separating and clearly presenting what was done in the quantitative and qualitative study – for example, how many participants in each study, what are the inclusion criteria in each study, instruments used in both studies. | Revised  Number of participants in the quantitative survey= 1180  22 FGD Total participants 172 older males and females)  Inclusion and exclusion criteria- (Line (225-234)  Adults aged 60 years and above and permanently living continuously in the selected communities for at least 12 months preceding the time of the study were eligible to participate in the study. The eligibility was based on the criteria used in a similar community-based survey conducted by Gomez-Olive et al. (2018) in South Africa [45]. However, older persons who had any form of incapacitation or health conditions such as dementia, speech or hearing disorders and other communication problems and could not personally provide information were excluded from the study. Proxies were not utilized for this study because the objective was to document the older person’s views about their preferences and ageing experience, which a proxy may not accurately capture. |
|  | What does the acronym LGA, which appears on line 174, mean? | Local Government Area (LGA) Fully Spelt line 165 |
|  | It is very important to describe how the qualitative data were analyzed – What is the procedure, what is the unit of analysis, etc. | Revised Line 267-282- |
|  | **Materials and methods** |  |
|  | Regarding the quantitative study, the variables analyzed and the instruments used to evaluate them must be presented | Line 298-324 |
|  | Results |  |
|  | In general, the quantitative results are well presented. The tables are clear, and the description is clear. | Noted |
|  | After line 247 (there is no longer any reference to the lines) it says: “Other factors associated with a desire to AIP among the rural participants were having owned homes, assistance at home and good self-rated quality of life (SRQoL)”. – How was quality of life assessed in this study? It is important to clarify this in the methodology part. | Participants attitude to ageing was assessed using the attitude to ageing questionnaire (AAQ). This measured the attitude in psychosocial, physical change and psychological domains. Participants were asked to rate their quality of life on a five-point Likert scale ranging from very satisfied to very bad. The responses were categorized as having good QoL (if their response to the question of Qol were “good” or “very good” with their Qol. Likewise, respondents were said to have poor QoL if they reported these to be “bad”, “very bad”, or “neither bad nor good”; with their QoL. Participants were asked to rate their health on a five-point Likert scale ranging from very good to very bad. The responses were categorized as having good self-rated health SRH (if their response were “good” or “very good”. Likewise, respondents were said to have poor SRH if they reported these to be “bad”, “very bad”, or “neither bad nor good”; The respondent’s level of functioning was measured using the 12- item World Health Organization Disability Assessment |
|  | In general, the qualitative results are quite complete and in-depth. I appreciate the effort to have made this presentation so exhaustive. However, I suggest that the presentation of the results be reduced to representative data, so that the essence of the results is not lost. I suggest that this section be reorganized, in order to make the results clearer and more comprehensive and to make reading more fluid and easier. - The themes, categories and sub-categories that resulted from the qualitative analysis should be presented explicitly in the text, along with their meaning and more representative examples. | Done (Table 4) |
|  | - The transcripts to illustrate the categories and sub-categories should be shorter and the most representative. When there are too many transcripts, the reader gets lost in the text and misses the essence of the results. | Done (Table 4) |
|  | **Discussion** |  |
|  | The discussion articulates well the data from qualitative and quantitative studies and the results with evidence from other studies. I suggest that after the objectives have been reformulated in the introduction, they are addressed in the discussion, showing the extent to which this study has allowed to meet them. | Revised |
|  | **Reviewer #2** |  |
|  | The paper “Ageing in place or stuck in place: Long-term care preferences of community-dwelling older persons in a low resource country in Sub Saharan Africa”, seems to me to be very relevant, as research on ageing is predominant in Euro-American countries. However, in terms of content it is important to distinguish "Aging in place" from "Long-term care" in the introduction, considering that in a continuum of care we are practically in opposite poles. This problem is present in the whole article, from introduction, to method, to the Table titles in the Results section. Therefore, when problematizing the topic under investigation, it is necessary to precisely define the mains concepts to clarify study goals. The analysis of some systematic reviews in the field may help to clarify the subject (Rosenwohl-Mack et al., 2020; Ahn, 2017). | The manuscript has been revised to reflect strictly the preferred care setting for older persons. i.e Aging in place within the home or community compared to other options such as moving in with the child, relatives, or care by religious organizations or within institutional settings or other |
|  | **Methods** |  |
|  | Regarding the method section, the mixed-methods design is particularly interesting. Simply, because it is a demanding methodology, it is essential to present in sufficient detail each phase of the study (first Quantitative, followed by Qualitative). | Revised to reflect what was done in the qualitative and quantitative aspects. |
|  | Quantitative - the sample and sampling are very clear. On the other hand, data collection instruments and procedures are practically omitted. For example, there is reference to a Questionnaire. What kind of questionnaire? With open questions? Closed Questions? How many items? On what topics? | A semi-structured questionnaire was developed reflected the study objectives and consisted of the following sections:.  Sociodemographic characteristics and family dynamics  Current health status and selected health risk behaviours  Attitude to ageing  Assessment of Quality of life  Assessment of self-reported functioning and disability |
|  | The tables in the results section provide some information regarding questions/items, but the information available to readers is insufficient. For example, in the logistic regression, it is necessary to indicate which variables are constants of the regression model and inclusion criteria, if applicable. | Variables that are already proven in the literature to be related to the outcome (preference for AIP) were included in the regression model and fitted for the rural, urban, and total population at a 5% statistical significance level. SRH was excluded because of collinearity. The final model was presented. |
|  | Qualitative - the use of Focus Group is very interesting. There is some information, but important details are missing. For example, how were the task distributed within the team? Who led the focus group? Who was responsible for the dynamics of the group? How was the problem of simultaneous speech solved both in the management of the group and later in the transcription? As for the results, the sequence of presentation seems to be clear: (1st) quantitative data and (2) qualitative data. However, there are some aspects that need to be improved Quantitative data: Verify tables designations in view of the objectives of the study, i.e., if “aging in place” or "long-term care". | The distribution of tasks for the FGD has been spelt out.  The facilitator of each discussion was responsible for the dynamics of the group.  Ground rules were set to avoid simultaneous speech and the facilitator ensured everyone had an opportunity to voice their opinions without interruption or overruling  The objective of the study was the preferred setting for care. i.e ageing in place (home or community) |
|  | Qualitative data: in quotations from the focus group, besides age group, authors should add the participants’ age. If we have more information about the participants (age, sex, area of residence, etc.) we can more easily observe trends in the variables under study and especially perform a more dynamic interpretation of quantitative and qualitative results. | Age, Sex and Area of residence already included in the quotes. The groups were stratified based on Sex (Male, Female) ,age ( <70 and ≥70 years) and Area of residence (rural urban).  Each quote is thus labelled in that format. For example**, Male FGD_ 70 years and above_ Urban** |
|  | **Discussion and conclusion** |  |
|  | It is important to highlight the main findings (quantitative and qualitative) in order to discuss research trends. At the same time, the authors must clarify if the paper main contributions address "aging in place", "long-term care" or both. It is necessary to include study limitations | The main findings of the study has been summarized and included  The papers main contribution addresses Aging in Place  Study limitations included |
|  | Other aspects to consider |  |
|  | Imprecisions, as the number of participants in the method section – Sampling Technique, line 183 [Similar to previous studies, if the 58 [588?] selected respondent….]. Equally, | Corrected |
|  | in-text citations do not always follow the same reference norms throughout the paper | References have been corrected |
|  | Even though superscript numerals predominate, there are cases with publication year. It is recommended to correct. Example Introduction, line 96 [For instance, Greenfield (2016=, in a qualitative study among older persons….] | Corrected |
