# Supplementary material for: Ageing in place or stuck in place: Preferred care setting for community-dwelling older persons in a low-resource country in Sub Saharan Africa
Source: PLoS One. 2023 Oct 16;18(10):e0292939. doi: 10.1371/journal.pone.0292939 (PMC10578603; doi:10.1371/journal.pone.0292939)
Supplement: Attachment — Submitted filename: Response to Reviewers Comments_ resubmission_20230918.docx [file pone.0292939.s002.docx]

| **Serial Number** | **Reviewers Comment** | **Response** | **Page Number** |
| --- | --- | --- | --- |
| 1. | The methods section needs further adjustments because the presentation sequence used for the research plan (qualitative approach followed by quantitative approach) is precisely the reverse of what is shown in the results section. | Methods section has been edited in the suggested order  -first, the quantitative approach  -then the qualitative approach (i.e., focus group). | Pages 12-18 |
| 2. | There is scattered information that needs to be associated with the respective methodological approach. | Noted scattered information has been associated with the respective methodological approach. | Pages 12-18 |
|  | I suggest that the research plan, in the methods section, is presented in the same order: (1) first, the quantitative approach (sample, data collection protocol, analytic strategy, and other information deemed convenient); (2) only then the qualitative approach (i.e., focus group). | The research plan, in the methods section, has been edited and presented in the suggested order: (1) first, the quantitative approach (sample, data collection protocol, analytic strategy, and other information deemed convenient); (2) then the qualitative approach (i.e., focus group). | Pages 12-18 |
|  | All the information that concerns each study approach should be associated with the approach it refers to. In other words, a better organization of contents in the methods section will make the research plan more comprehensible | Contents in the methods have been re-organised | Pages 12-18 |

Dear Sir,

Thank you for the detailed review of my manuscript. Please find below the response to the comments made.

Thank you.

E. Cadmus
